# Adaptive Response of *Saccharomyces* Hosts to *Totiviridae* L-A dsRNA Viruses Is Achieved through Intrinsically Balanced Action of Targeted Transcription Factors

**DOI:** 10.3390/jof8040381

**Published:** 2022-04-09

**Authors:** Bazilė Ravoitytė, Juliana Lukša, Ralf Erik Wellinger, Saulius Serva, Elena Servienė

**Affiliations:** 1 Laboratory of Genetics, Institute of Botany, Nature Research Centre, Akademijos Str. 2, 08412 Vilnius, Lithuania; juliana.luksa@gamtc.lt; 2 Department of Biochemistry and Molecular Biology, Institute of Biosciences, Vilnius University, Saulėtekio Al. 7, 10257 Vilnius, Lithuania; saulius.serva@gf.vu.lt; 3 Genome Stability Department, Andalusian Center for Molecular Biology and Regenerative Medicine CABIMER, Universidad de Sevilla-CSIC, Avda. Américo Vespucio 24, 41092 Seville, Spain; ralf.wellinger@cabimer.es; 4 Department of Chemistry and Bioengineering, Vilnius Gediminas Technical University, Saulėtekio Al. 11, 10223 Vilnius, Lithuania

**Keywords:** *Totiviridae*, dsRNA, transcription, yeast, *Saccharomyces cerevisiae*, *Saccharomyces paradoxus*

## Abstract

*Totiviridae* L-A virus is a widespread yeast dsRNA virus. The persistence of the L-A virus alone appears to be symptomless, but the concomitant presence of a satellite M virus provides a killer trait for the host cell. The presence of L-A dsRNA is common in laboratory, industrial, and wild yeasts, but little is known about the impact of the L-A virus on the host’s gene expression. In this work, based on high-throughput RNA sequencing data analysis, the impact of the L-A virus on whole-genome expression in three different *Saccharomyces paradoxus* and *S. cerevisiae* host strains was analyzed. In the presence of the L-A virus, moderate alterations in gene expression were detected, with the least impact on respiration-deficient cells. Remarkably, the transcriptional adaptation of essential genes was limited to genes involved in ribosome biogenesis. Transcriptional responses to L-A maintenance were, nevertheless, similar to those induced upon stress or nutrient availability. Based on these data, we further dissected yeast transcriptional regulators that, in turn, modulate the cellular L-A dsRNA levels. Our findings point to totivirus-driven fine-tuning of the transcriptional landscape in yeasts and uncover signaling pathways employed by dsRNA viruses to establish the stable, yet allegedly profitless, viral infection of fungi.

## 1. Introduction

Persistent dsRNA viruses in yeasts are usually associated with the killer phenotype of the host cell [1,2,3,4]. *Totiviridae* L-A virus is an essential component of the dsRNA-based killer system. It encodes the major coat protein (Gag) and RNA-dependent RNA polymerase (Pol), proteins essential for the replication, encapsidation, and maintenance of both the L-A and the M satellite virus, which provide the killer phenotype to the host cell [1]. While dsRNA viruses of fungi often promote hypo- or hypervirulence or a killer phenotype [5,6,7,8], infection with L-A alone seems to be symptomless in yeast [9]. It appears as a result of long-lasting coevolution, as evidenced by the enormous quantity of 4.6 kb-sized L-A dsRNA, which is comparable to that of cellular rRNAs [10].

Several variants of *Totiviridae* L-A have been described in closely related *Saccharomyces cerevisiae* and *S. paradoxus* species [2,3,11,12,13,14,15], and the list of known L-A virus hosts is further expanding [16]. Despite the high average identity of nucleotide sequences, L-A viruses show complex relationships of compatibility with M satellites [14,15,17,18]. The L-A virus seems to be less challenging for the host than satellite M, since only a few genes were previously described to be crucial for L-A maintenance [17]. The factors and mechanisms contributing to L-A virus maintenance are less studied, but L-A dsRNA tends to be more resilient to elimination than M dsRNA [19]. A recent study showed that the overproduction of trimmed L-A capsid protein results in the complete elimination of the virus genome, and does not depend on the L-A type, suggesting similar mechanisms of L-A maintenance in non-killer yeasts [18].

At least several host genes and metabolic conditions are linked to the replication and maintenance of the L-A virus. L-A replication has been reconstructed in vitro and is uniformly believed to occur in cytoplasm via a conservative transcription mechanism [17,20]. N-terminal Gag acetylation by NatC N-acetyltransferase, encoded by the *MAK3*, *MAK10*, and *MAK31* genes, and other yet-unknown host factors are required for the assembly of L-A virus-like particles [17,21,22,23]. These undetermined factors are likely essential for host viability or substitute one another, thereby complicating their identification. The maintenance of yeast dsRNA viruses is very sensitive to changes in ribosome structure; thus, killer yeasts serve as a convenient model to study the function of ribosomal proteins [24]. Ribosomes are important for L-A propagation [25], and mutations of *RPL3*, *RPD3*, *SIN3*, *SAP30*, *RPL41A*, and *RPL41B* elevate the efficiency of programmed −1 ribosomal frameshifting and often result in a decrease or even in a loss of viral dsRNA(s), mostly affecting satellite M [24,26,27]. A lower L-A dsRNA copy number and the amount of Gag protein were observed in cells with disrupted *RPL4A*, *RPL4B, MAK11*, and *MAK16* genes, which are involved in the biosynthesis of 60S ribosomal subunits [25]. It was suggested that 60S ribosomal subunits are required for the sufficient translation of L-A capsid proteins; however, there were also exceptions, likely related to the differences in the L-A mRNA sequences between L-A variants [25]. Respiration-deficient non-killer yeast mutants lacking mitochondrial pore protein accumulate large amounts of virus-like particles and L-A dsRNA during adaptation to growth on glycerol [28]. Ethanol-grown yeast cells contain higher levels of viral dsRNA than glucose-grown cells [29], which may be related to the observation that *MAK10* is repressed by glucose [30]. It was also proposed that the L-A virus and mitochondrial DNA compete for Mak10 protein [30]. Double-stranded RNA viruses were shown to mask distinct chromosomal mutations and, in some instances, they were affected by a variant of the mitochondrial genome, suggesting that non-chromosomal elements can interact to alter the host phenotype [31].

L-A virus propagation is controlled or inhibited by systems found in yeast cells. Nascent L-A mRNA has neither cap nor polyA tails [32]; thus, its 5′-end is prone to degradation by exonuclease Xrn1/Ski1, whereas cytoplasmic exosome degrades mRNA, which lacks a polyA tail in the 3′-end [12,33]. Xrn1 is involved in the regulation of various cellular processes, including meiosis [34], filamentous growth [35], RNA turnover [36], control of telomere length [37], respiration [38], and autophagy [39]. The action of the exosome is linked to the SKI complex, in which Ski2, Ski3, Ski7, and Ski8 proteins are required for the proper identification of exosome targets (see [40] for review). The disruption of any of these *SKI* genes enables higher levels of viral dsRNA; in case of the presence of M virus, markedly higher production of killer toxin, leading to the so-called “superkiller” phenotype [33]. To avoid these mechanisms, the virus has developed protection strategies. The cap-snatching mechanism for the translation of L-A proteins was proposed [41]. The ends of L-A positive strands are 5′ diphosphorylated by yet-unknown proteins, even though the triphosphatase and kinase activities have been associated with L-A virus-like particles [42,43]. Diphosphorylation is essential for viral transcription and acts like a self-identity tag of viral RNA [43]. Mitochondrion-related nuclease *NUC1* and mitochondrial porin-encoding *POR1/2* genes negatively regulate the copy number of L-A dsRNA [28,44,45]. This regulation can be crucial for the survival of sporulating cells [45].

Several studies have highlighted the changes in the L-A virus level in yeast cells upon different metabolic conditions and sporulation [28,44,45]. Recently, host gene transcription in yeasts hosting or cured of dsRNA viruses was examined [46,47,48,49]. Transcriptome studies mainly involve wild-type killer cells as a reference, making the impact of L-A and M viruses overlapping, since killer cells possess two types of dsRNA. While the incidence of killer viruses in the *S. cerevisiae* and *S. paradoxus* strains is estimated to be relatively infrequent, L-A dsRNA is nevertheless prevalent in various wild, industrial, clinical, and laboratory yeasts [50]. Biological sensing of the maintenance of the L-A virus in non-killer yeasts, therefore, remains a long-standing challenge in the field of yeast dsRNA viruses.

In this study, we analyzed the transcriptional responses in L-A dsRNA maintaining non-killer *S. cerevisiae* and *S. paradoxus* yeasts. We, therefore, strove to analyze the transcriptional pattern of L-A dsRNA maintaining non-killer *S. cerevisiae* (M437 harboring L-A-lus) and *S. paradoxus* (AML-15-66 Spo and Pet harboring L-A-66) yeasts based on high- throughput RNA-seq data as compared to dsRNA-free cells, respectively [47,49]. Cured *S. cerevisiae* cells show the same phenotype as the wild-type cells, except for the absence of the killing ability, while cured *S. paradoxus* cells gain either respiration-deficient (named Pet) or increased sporulation frequency (named Spo) phenotypes [47,49]. Interestingly, the moderate by intensity, yet broad, transcriptional reprogramming of a host cell upon L-A virus infection is rather yeast strain-specific. Nevertheless, a common hallmark is the transcriptional modulation of genes linked to various stress responses. We found that mutant cells lacking the transcription factors involved in stress response activation are prone to a significant increase or decrease in the L-A dsRNA levels. Thus, our findings shed new light on the host factors beyond the precious balance of L-A maintenance.

## 2. Materials and Methods

### 2.1. Gene Expression Data

Processed transcriptomics data were obtained from the Gene Expression Omnibus (GEO) repository under accession numbers GSE100290 [47] and GSE153308 [49]. From the GSE100290 dataset, providing gene expression data in cells derived from *S. cerevisiae* M437 [kil-K2] [51], three replicates of cells with L-A-lus virus and three virus-free samples were collected. From GSE153308 the dataset, providing transcriptomic data of cells derived from *S. paradoxus* AML-15-66 [kil-K66] [2], two different (altered-sporulation and respiration-deficient) cell types (three replicates of cells with L-A-66 virus and three virus-free samples for each cell type) were analyzed. All datasets exploited the same yeast growth, RNA extraction conditions, and overall experimental design. Yeast cells were grown in standard liquid complete medium for yeast growth YPD at 25 °C with shaking at 250 rpm. The total RNA for high-throughput RNA sequencing was extracted from cells in the exponential growth phase (OD_600_ = 0.5–0.6) using GeneJET RNA Purification Kit (Thermo Fisher, Vilnius, Lithuania), according to the Yeast Total RNA Purification Protocol provided by the manufacturer [47]. Detailed descriptions of the experiments that led to the generation of processed transcriptomics data in the GSE100290 and GSE153308 datasets are provided in the following publications [47,49].

### 2.2. Identification of Differently Expressed Genes (DEGs)

The mean expression values were used to determine differential expression by implementing the “exact test” of Robinson and Smyth [52] by applying the DGE tool in CLC Genomics Workbench v12.0 (CLC Inc., Aarhus, Denmark; http://www.clcbio.com). Gene expression was considered significant if differently expressed genes (DEGs) had a fold-change of ≥1.5 and FDR-corrected *p*-value of ≤0.05 [53]. Two group comparisons were made between M437[L−M−] vs. M437[L+M−]; Spo[L−M−] vs. Spo[L+M−]; and Pet[L−M−] vs. Pet[L+M−]. Descriptions of the gene products of DEGs were obtained using YeastMine (http://yeastmine.yeastgenome.org, accessed on 15 July 2021) data search and retrieval tool [54] (Table S1).

**Figure 1:**
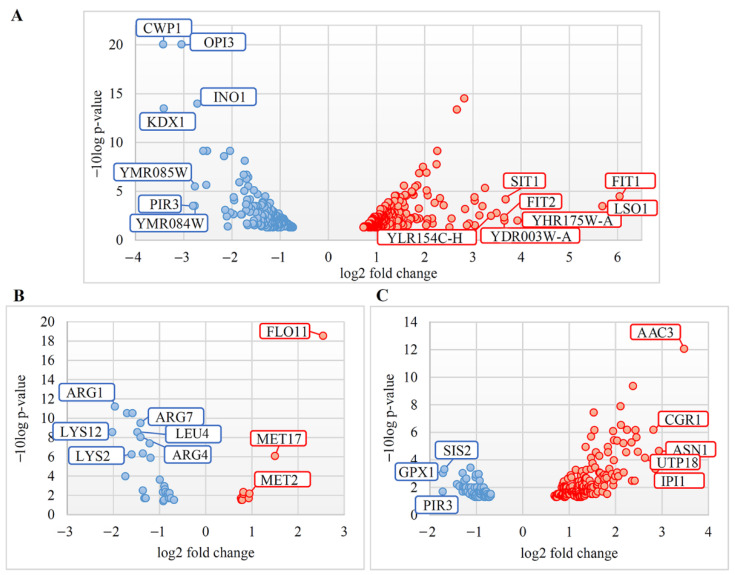
Volcano Plots for differential gene expression in *S. cerevisiae* M437[L] and two *S. paradoxus* AML-15-66 strains—Spo[L] and Pet[L]. Scattered points represent differently expressed genes (DEGs): red, upregulated; blue, downregulated. The x-axis represents the fold-change of the DEGs, whereas the y-axis indicates the log odds—the probability that the differential expression of a gene has statistical significance. DEGs in (**A**) M437[L], (**B**) Spo[L], and (**C**) Pet[L] cells are represented. M437[L], Spo[L], Pet[L]: DEGs in solely L-A-maintaining cells with reference to dsRNA-free cells of the same lineage.

Volcano plots showing the relationship between the *p*-value and the magnitude of the difference in the fold-change of the samples were counted and visualized in Microsoft Excel (Figure 1).

**Figure 2:**
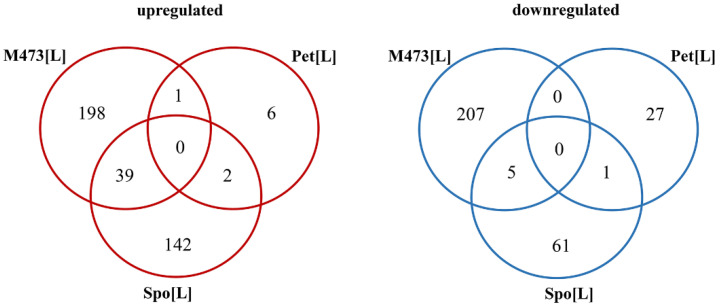
Venn diagrams representing the numbers of differently expressed genes (DEGs) in *S. cerevisiae* M437[L], and *S. paradoxus* AML-15-66 Spo[L] and Pet[L] cells. M437[L], Spo[L], and Pet[L]: differently expressed genes in solely L-A maintaining cells with reference to dsRNA-free cells of the same lineage.

Venn diagrams comparing the DEGs of different cells (Figure 2) were generated by using an online tool for the calculation and drawing of custom Venn diagrams (http://bioinformatics.psb.ugent.be/webtools/Venn/, accessed on 4 February 2021).

### 2.3. GO Pathway Enrichment Analysis

To characterize the relationships between genes, Gene Ontology (GO) analysis was performed. GO TermFinder (http://go.princeton.edu, accessed on 7 September 2021) was used to detect the most significant GO terms [55]. Gene products were annotated by the “Biological processes”, “Molecular function”, and “Cellular component” aspects. The obtained results were analyzed, the statistical significance of each annotation was calculated using hypergeometric distribution, and Benjamini and Hochberg false discovery rate correction was conducted. Results with a *p*-value of <0.05 were represented (Table S1). The fold-enrichment (F.E.) was calculated by comparing the background frequency of genes annotated to the GO term, with the sample frequency representing the number of input genes that fell under the matching GO term.

### 2.4. Essential Genes

Essential genes were selected based on information provided in the SGD (Saccharomyces Genome Database [56], http://www.yeastgenome.org, accessed on 22 July 2021). In our study, 506 yeast genes were considered as essential. A total of 474 essential genes were selected with reference to documented evidence provided by experimental data from classical genetics, referred to in the SGD. A gene was assumed as being essential for yeast viability if its complete elimination from the genome was reported to provide lethality in at least one *S. cerevisiae* yeast strain (CEN.PK, D273-10B, Y55, JK9-3d, S288C, SEY6210, Sigma1278b, SK1, W303, or others). In addition, 32 genes performing essential functions according to the description provided by SGD were also discussed as essential. The systematic gene names of essential genes are marked with different colors in the lists of DEGs in Table S1.

### 2.5. Determination of Transcriptional Regulators

Transcription factors (TF) linked to the transcription of DEGs were deducted using the tools available in the YEASTRACT (Yeast Search for Transcriptional Regulators AND Consensus Tracking, http://www.yeastract.com, accessed on 17 November 2021) repository [57]. The activity of each TF was predicted by the number of documented targets in the corresponding datasets considering only documented direct regulatory associations based on expression evidence. Lists of upregulated genes were only searched for the targets of transcriptional activators, while those of downregulated genes were only searched for the targets of transcriptional repressors. DEGs were ranked based on the percentage of genes in the list and in the YEASTRACT database regulated by each TF. The *p*-value was calculated using the hypergeometric test. A full list of TFs is provided in Table S2, where TFs are ranked from the highest to the lowest-confidence-level regulatory associations.

### 2.6. Strains and Media

To evaluate the effects of gene transcription factors on the L-A dsRNA levels, *Saccharomyces cerevisiae* strains (BY4741 background, MATa; his3Δ1; leu2Δ0; met15Δ0; and ura3Δ0) were employed. To study the effects of single transcription factor deletions, strains from the *S. cerevisiae* nonessential gene deletion collection (YKO) with single ORFs replaced by the KanMX4 module were tested. These strains were purchased from Thermo Scientific Molecular Biology (Lafayette, CO, USA). Yeast knockout library (YKO) strains were grown in standard complete medium for yeast growth (YPD) (1% yeast extract, 2% peptone, 2% dextrose; 2% agar was added for solid medium) for 16 h at 25 °C with shaking at 250 rpm.

For transcription factor overexpression experiments, strains from Yeast GST Collection were used (Dharmacon, Lafayette, CO, USA). The overexpression of plasmid-encoded ORFs was under the control of the GAL1/10 promoter. These strains were grown on SD medium (0.67% yeast nitrogen base, L-leucine (60 µg/mL), L-methionine (10 µg/mL), and L-histidine (10 µg/mL), with 2% agar for solid medium) supplemented with 2% glucose for recovery. To induce the overexpression of selected transcription factors, freshly grown cells were grown in liquid SD medium with the required amino acids and 2% galactose (instead of glucose) for 16 h at 25 °C with shaking at 250 rpm.

### 2.7. Total RNA Extraction and Evaluation of L-A dsRNA Content Changes

Yeast mutant strains with single deletions of transcription factors (except for Met4p, Cyc8p, and Rsc30p) with the highest confidence level (marked in green, Table S2) were subjected to total RNA isolation. Only strains overexpressing selected transcription factors that were significantly altering the L-A dsRNA levels upon deletion were analyzed.

The total RNA was extracted as described in a previous study [47]. The mutant and wild-type strains were grown in YPD medium, whereas strains with plasmid-encoded genes were grown in SD medium with the required amino acids and 2% galactose.

The extracted total RNA was analyzed by 1% agarose gel electrophoresis. The dsRNA amount of L-A virus for each sample was normalized by the 18S rRNA levels and compared to the values of the control samples that were grown in the same medium. Only the strains with the highest difference in the L-A dsRNA levels were considered to be significant.

## 3. Results

### 3.1. Transcriptional Response in L-A dsRNA Maintaining S. cerevisiae and S. paradoxus Cells

To better understand the role of L-A dsRNA virus for a host strain, we compared the changes in gene expression between *S. cerevisiae* M437 and *S. paradoxus* AML-15-66 cells possessing solely L-A dsRNA in relation to the corresponding dsRNA-free cells (see Table S1 for a full list of DEGs). In addition to focusing on different L-A viruses found in these yeasts (L-A-lus in M437 and L-A-66 in AML-15-66), distinct states of metabolism were addressed: *S. cerevisiae* M437 (harboring single L-A-lus virus; thus, named M437[L]) features regular glucose-dependent metabolism, while *S. paradoxus* AML-15-66 cells (bearing solely L-A-66 are named Spo[L] and Pet[L]) indicate altered sporulation and respiration-deficient (*petite)* phenotypes, as described in the previous study [49].

The presence of L-A dsRNA leads to moderate transcriptional alterations in the examined strains. Volcano plots (Figure 1) represent the confidence levels and fold-changes of DEGs in M437[L], Spo[L], and Pet[L] cells. Approximately 88% of the DEGs in *S. cerevisiae* M437[L] and 79% of those in *S. paradoxus* Spo[L] cells were altered up to three times. The expression of 79% of the downregulated genes in *S. paradoxus* Pet[L] cells was also altered less than three-fold, while 78% of the positively regulated genes were upregulated up to two-fold (Table S1).

Among the most upregulated genes in M437[L] cells, genes encoding mannoproteins related to iron transport (*FIT1*, -*2*, -*3*), other proteins related to iron (*LSO1*, *SIT1*), and putative proteins of unknown functions (*YHR175W-A*, *YDR003W-A*, and *YLR154C-H*) (Figure 1 and Table S1) were identified. The most downregulated genes in M437[L] cells were those encoding the cell wall proteins (*CWP1*, *PIR3*), protein kinase (*KDX1*), and proteins involved in cell wall and membrane biosynthesis (*OPI3*, *YMR084W*, *YMR085W*, and *INO1*).

The genes most upregulated in the Spo[L] cells were *AAC3* (encoding mitochondrial ADP/ATP translocator), *ASN1* (coding for asparagine synthetase), and those related to ribosome biogenesis (*IPI1, CGR1,* and *UTP18*). The *PIR3* (encoding cell wall protein)*, GPX1* (coding for glutathione peroxidase), and *SIS2* (encoding a negative regulatory subunit of protein phosphatase 1) genes were the most downregulated in Spo[L] cells (Figure 1 and Table S1).

The most upregulated genes in Pet[L] cells were *FLO11* (encoding flocculin), *MET17,* and *MET2* (both involved in the metabolism of methionine). The most downregulated genes in Pet[L] cells were related to the biosynthesis of amino acids (e.g., *LYS2*, *-12*, *ARG1*, *-4, -7*, and *LEU2*) (Figure 1 and Table S1).

Overall, gene transcription was the most altered (450 genes) in M437[L] cells, less (250 genes) in Spo[L] cells, and the least (37 genes) in Pet[L] cells (Figure 2 and Table S1). The numbers of up- and downregulated genes in M437[L] cells were similar, with 238 genes being up- and 212 being downregulated. Approximately two-thirds (183 genes) of the DEGs in Spo[L] cells were upregulated, and about one-third (67 genes) were downregulated. An inverse distribution of the gene number ratio was observed in Pet[L] cells, where about one-third of the DEGs were upregulated (nine genes) and two-thirds were downregulated (28 genes) (Table S1). The gene transcription patterns were highly different between each cell type; there were no DEGs shared between all three strains, neither upregulated nor downregulated (Figure 2). Only *LYS12* was differently expressed in all three strains; however, it was upregulated in M437[L] and Spo[L], but downregulated in the Pet[L] cells.

The highest number of mutually regulated DEGs was between M437[L] and Spo[L] cells (Figure 2). The majority of spotted genes were linked to the biosynthesis of ribosomes and amino acids. Of the 48 DEGs shared between the M437[L] and Spo[L] cells, 44 were mutually regulated. Thirty-nine of these genes were upregulated and mostly related to ribosome biogenesis (*BUD22, DIP2, NSR1, PUF6, RCL1, RPA135, RRP12,* and *RRS1*) and aspartate-family amino acids (*AAT1, ACO2, ASN1, IRC7,* and *ILV1*). In M437[L] and Pet[L] cells only, the expression of the *MIG2* gene, encoding a protein involved in glucose-dependent gene repression [58], was upregulated. On the other hand, the expression of *ZRT1* and *MET17* genes, which are linked to zinc uptake [59] and the biosynthesis of sulfur amino acids [60], respectively, were mutually upregulated in Spo[L] and Pet[L] cells.

There was little similarity between the profiles of downregulated genes in the tested cells (Figure 2). The *OPI3, PIR3, EHT1, GPX1,* and *RCN1* genes were negatively regulated in both M437[L] and Spo[L] cells, and most of these genes are linked to the metabolism of phospholipids. Only the *SSA4* chaperone-encoding gene was mutually downregulated in *S. paradoxus* Spo[L] and Pet[L] cells. *MAK21*, involved in ribosome biogenesis and dsRNA maintenance, was upregulated in M437[L] cells.

### 3.2. Gene Ontology Analysis of DEGs in Solely L-A dsRNA Maintaining Cells

**Figure 3:**
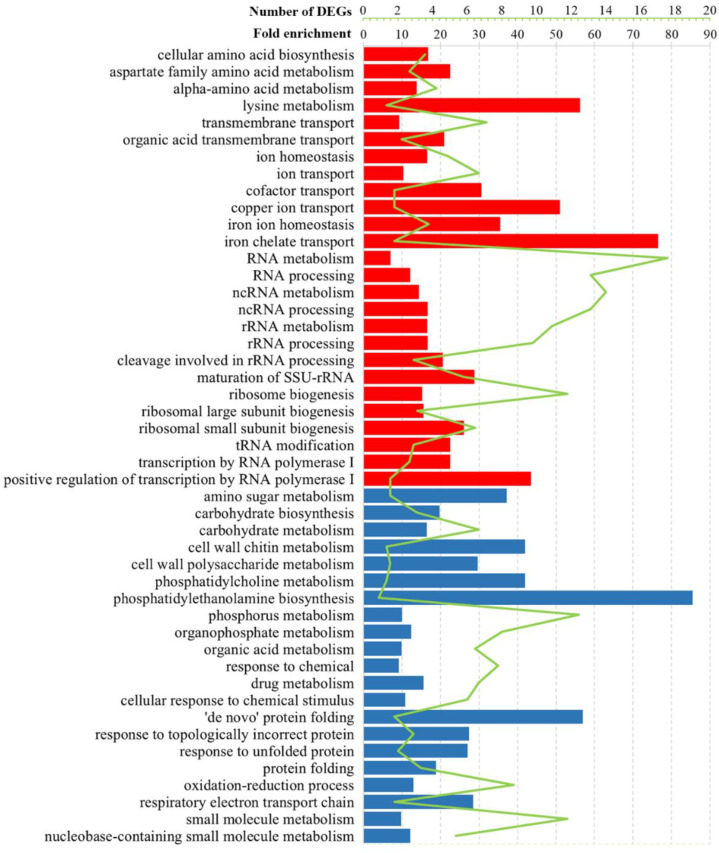
Selected statistically significant enriched gene ontology terms associated with the biological processes of altered transcription genes in *S. cerevisiae* M437[L] cells. The green line represents the number of DEGs, whereas the bars indicate the fold-enrichment (F.E.) values. Upregulated genes, red; downregulated genes, blue. F.E. values were calculated by dividing the frequency of the specific gene cluster by the total frequency for each GO term according to the data presented in Table S1.

**Figure 4:**
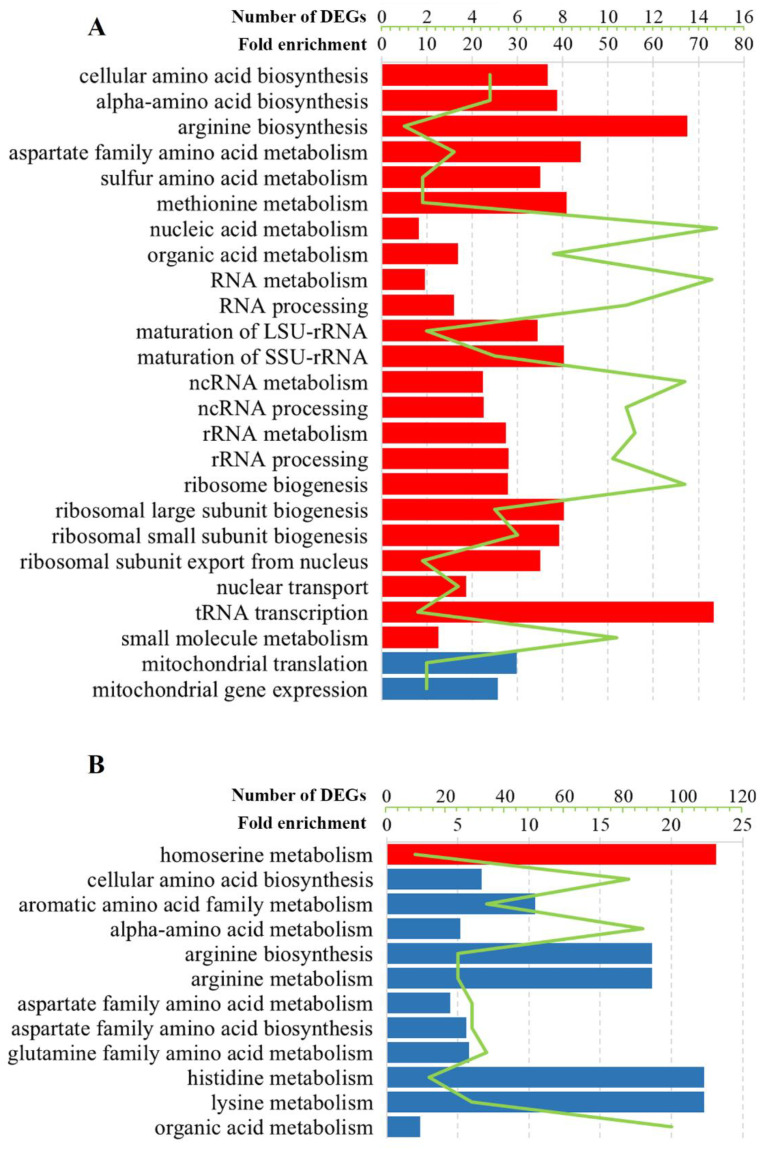
Selected statistically significant enriched gene ontology terms associated with the biological processes of altered transcription genes in (**A**) *S. paradoxus* AML-15-66 Spo[L] and (**B**) Pet[L] cells. The green line represents the number of DEGs, whereas the bars indicate fold enrichment (F.E.) values. Upregulated genes, red; downregulated genes, blue. F.E. values were calculated by dividing the frequency of a specific gene cluster by the total frequency for each GO term according to the data presented in Table S1.

GO analysis of DEGs revealed similarities and differences between *S. cerevisiae* M437[L] and *S. paradoxus* Spo[L] cells and Pet[L] cells. The analysis of DEGs showed nine mutual GO process terms and one GO function term among the three cell types (Figure 3 and Figure 4, Table S1). Table S1 contains a complete set of GO process, function, and localization analysis results, as well as the calculated fold-enrichment (F.E.) values. The small-molecule, oxoacid, organic, and carboxylic acid metabolic processes were enriched by downregulated genes in M437[L] and upregulated genes in Spo[L] and Pet[L] cells (Table S1). The catalytic activity function was represented by negatively regulated genes in M437[L] and Pet[L] cells and by upregulated genes in Spo[L] cells.

The regulation of genes involved in the metabolism of amino acids was altered in all cell types (Figure 3 and Figure 4, Table S1). Positively regulated genes in M437[L] and Spo[L] cells and negatively regulated genes in Pet[L] cells were related to the biosynthesis and metabolism of the aspartate family and alpha-amino acids (Table S1). Lysine metabolism was represented by upregulated genes in M437[L] cells and by downregulated DEGs in Pet[L] cells (F.E. of 12.5 and 107.0, respectively). Arginine biosynthesis was associated with genes downregulated in Pet[L] and upregulated in Spo[L] cells (F.E. of 89.5 and 13.5, respectively). The metabolism of sulfur amino acids and methionine (F.E. of 7.0 and 8.2, respectively) was upregulated in Spo[L] cells. Genes related to the metabolism of the histidine, aromatic, and glutamine amino acid families (F.E. of 107.0, 50.0, and 27.8, respectively) were downregulated in Pet[L] cells.

RNA-related processes were represented by upregulated genes in M437[L] and Spo[L] cells (Figure 3 and Figure 4, Table S1). DEGs related to RNA processing and metabolism, including ncRNA and rRNA processing, the biogenesis of ribosomes (F.E. of 3.4 and 5.6), and transcription by RNA polymerase I (F.E. of 5.2 and 5.5) were upregulated in the M437[L] and Spo[L] cells. The binding of snoRNA (F.E. of 12.5 and 6.6) was also represented by positively regulated genes in these cells. The activities of RNA helicase (F.E. of 6.3), RNA methyltransferase (F.E. of 5.8), and tRNA modification (F.E. of 5.0) were enriched in M437[L] cells only (Table S1). The binding of mRNA (F.E. of 8.2) and the activity of RNA polymerase III (F.E. of 4.4) were enriched solely in Spo[L] cells. GO Cellular component enrichment analysis showed that many of the genes upregulated in the M347[L] and Spo[L] cells were associated with gene product localization in the nucleus (F.E. of 1.4 and 1.5), nucleolus (F.E. of 4.2 and 6.1), and pre-ribosome (F.E. of 5.6 and 8.9).

Gene ontology analysis revealed that, in M437[L] cells, upregulated genes were linked to ion transport, homeostasis, and transmembrane transport (Figure 3). The transport of cofactors, copper ions, iron ions, and siderophores was enriched (F.E. of 6.8, 11.3, 9.7, and 17.0, respectively) with positively regulated genes in M437[L] cells. Functional GO term analysis uncovered enrichment in the transporter and ferric-chelate reductase activities (F.E. of 2.2 and 17.0). Positively regulated genes in Pet[L] cells were related to homoserine metabolism (F.E. of 111.0), cofactor binding (F.E. of 11.5), and mitochondrial nucleoids (F.E. of 55.5); meanwhile, in Spo[L] cells, similarly to M437[L], upregulated DEGs were primarily involved in various RNA and amino acid processes (Figure 3 and Figure 4, Table S1).

Negatively regulated genes in M437[L] cells were related to the response to chemical stimulus (F.E. of 2.4) and unfolded proteins (F.E. of 7.0), cell wall (F.E. of 3.3), and respiratory electron transport chain (F.E. of 6.3) (Figure 3 and Table S1). Cell wall chitin and polysaccharides, UDP-N-acetylglucosamine metabolism, and the biosynthesis of phosphatidylcholine and phosphatidylethanolamine were enriched by downregulated genes in M437[L] cells (F.E. of 9.3, 6.6, 19.0, 9.3, and 19.0, accordingly). Downregulated genes in Pet[L] cells were mostly linked to amino acids, while those in Spo[L] cells were associated with the mitochondrial gene expression (F.E. of 5.1) and translation (F.E. of 6.0) (Figure 4). Genes related to mitochondria are represented by enriched structural constituents of the ribosome (F.E. of 13.4), mitochondrial ribosome (F.E. of 9.15), and mitochondrial intermembrane space (F.E. of 10.0) GO terms.

### 3.3. Differently Expressed Essential Genes

We found that the majority of DEGs altered upon L-A infection are not essential for yeast viability. Out of 506 essential genes, 88 were differently expressed in at least one type of solely L-A-maintaining cells. The transcription of 49, 46, and 1 essential gene was altered in the M437[L], Spo[L], and Pet[L] cells, respectively (Table S1). More than half of these genes are related to the biosynthesis of ribosomes (*DBP-*, *NOP-*, and *RPA-* genes) and tRNA transcription (*RPC-* genes, encoding RNA polymerase III subunits).

The majority of differently expressed essential genes were upregulated in the Spo[L] and M437[L] cells. There were seven shared essential genes of altered transcription between M437[L] and Spo[L] cells, and all were upregulated. Only *CAB1* was involved in coenzyme A biosynthesis, while others (*DIP2, RCL1 RPA135, RRS1, SPB1,* and *UTP18*) were related to ribosomes. *GGC1*, which is essential for mitochondrial genome maintenance and important for mitochondrial iron transport, was upregulated in M437[L] and downregulated in Pet[L] cells. Upregulated essential genes in M437[L] cells were also related to iron–sulfur proteins (*CFD1* and *YAH1*) and the nuclear envelope (*BRL1*), whereas those in Spo[L] cells were involved in ergosterol biosynthesis (*ERG1, -11*), the composition of RNA polymerases (*RPA-, RPB8, RPO26, RPC*- genes), pentose phosphate pathway (*RKI1*), and tRNA synthesis (*WRS1*). *RLI1*, encoding an essential iron–sulfur protein required for ribosome biogenesis, was upregulated in Spo[L] cells. *MAK5* was upregulated in M437[L] cells.

Only four essential genes (*HSK3, EAP1, UGP1,* and *MIA40*) were negatively regulated in Spo[L] cells. Downregulated essential genes in M437[L] cells were related to the folding, modification, and transport of proteins (*SSC1, HSP60, HSC82, PDI1, VPS17, UFD1,* and *CAR2*), and cell wall biogenesis (*DFG5* and *PCM1*). RNA exonuclease encoding *USB1* was the most downregulated essential gene in M437[L] cells.

### 3.4. Transcription Factors Regulating DEGs in Solely L-A-Infected Cells

**Figure 5:**
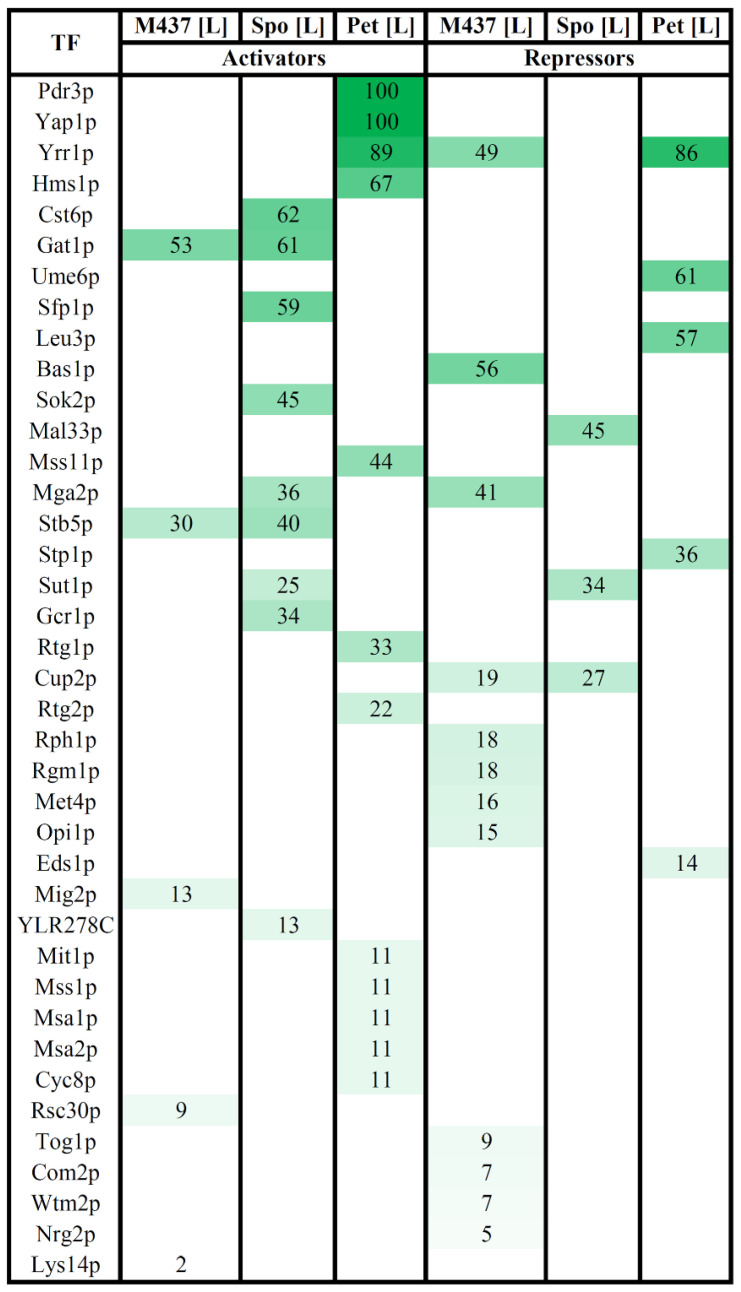
Associations between differently expressed genes (DEGs) and their documented regulators in *S. cerevisiae* M437[L], and *S. paradoxus* AML-15-66 Spo[L] and Pet[L] cells. Upregulated genes were only searched for transcriptional activators, while downregulated genes were only searched for transcriptional repressors using the tools available in the YEASTRACT database. TF: transcription factor; M437[L], Spo[L], Pet[L]: DEGs in solely L-A maintaining cells with reference to dsRNA-free cells of the same lineage. The impact of each TF was predicted based on the percentage of targets in each dataset only considering documented expression evidence. The percentage of genes regulated by each TF in each dataset of up- or downregulated genes is presented in the table. Only selected highest-confidence-level regulatory associations are presented. The higher green color intensity indicates the higher percentage of regulated genes. The full list is available in Table S2.

To assess the possible role of transcription factors (TFs) in the global transcriptional alterations in L-A virus maintaining cells, we employed YEASTRACT analysis tools [57]. We aimed to identify which TFs could be the key regulators (activators or inhibitors) of gene expression in virus-infected cells. We inspected the documented transcription activators of upregulated genes and inhibitors of downregulated genes in solely L-A-maintaining cells in respect of dsRNA-free cells (Figure 5 and Table S2).

Our analysis revealed that 39 TFs regulated the expression of DEGs at the highest confidence level (Figure 5 and Table S2). Twenty-four TFs were associated with the activation of upregulated genes and 18 TFs were linked with the inhibition of downregulated genes (Figure 5). Out of these, three TFs were involved in both the up- and downregulation of DEGs, depending on the strain (Figure 5). A significant part of the TFs involved in the regulation of DEGs was associated with the response to various stresses (e.g., Yap1, Pdr3, Rtg1, Rtg2, Yrr1, Stb5, Mga2, Cst6, Sfp1, Eds1, Cup2, Wtm2, and Rsc30), response to nutrients (e.g., Mss11, Gat1, Gcr1, Mal33, Nrg2, and Mig2), and related to amino acids (e.g., Leu3, Stp1, Met4, and Lys14).

Only several TFs were related with different expressions of a large part of the DEGs. The expression of 53% of the upregulated DEGs in M437[L] cells can be induced by Gat1, and the highest number of downregulated genes (56%) can be associated with Bas1-mediated repression. The transcription of the majority of positively regulated genes of Spo[L] cells is related to the action of Cst6 (62%), Gat1 (61%), and Sfp1 (59%) TFs. The expression of the highest percentage (45%) of downregulated genes in Spo[L] can be repressed by Mal33. Up to 100% of the DEGs in Pet[L] cells can be differently expressed by TFs Yap1 (100% of upregulated genes), Pdr3 (100% of upregulated genes), and Yrr1 (89% of up- and 86% of downregulated genes).

Among the six datasets (up- and downregulated genes of each cell type) of DEGs in M437[L], Spo[L], and Pet[L] cells, the gene expression pattern in M437[L] and Spo[L] cells seemed to be the most similar in terms of both shared genes (Figure 2 and Table S1) and related TFs (Figure 5 and Table S2). TFs Stb5 and Gat1 may activate the expression of a part of the upregulated genes, while Cup2 may repress the expression of a fraction of the downregulated genes in M437[L] and Spo[L] and cells (Figure 5 and Table S2). TF Yrr1 may be important for controlling the downregulation of 49% of genes in M437[L] cells, and for the vast majority of DEGs in Pet[L] cells. Sut1 may have an impact on the expression of 25% of up- and 34% of downregulated DEGs in Spo[L] cells. Mga2 protein could activate the transcription of 36% of the upregulated genes in Spo[L] and repress 41% of the downregulated genes in M437[L] cells.

The expression of three genes encoding the transcription factors Bas1, Mig2, and Rsc30 was upregulated in M437[L] cells, and *MIG2* was also upregulated in Pet[L] cells. Bas1 is involved in the repression of 56% of the downregulated genes in M437[L] cells (Figure 5 and Table S2). TF Rsc30 regulates 9% of the upregulated genes in M437[L] cells. Mig2 is associated with the activation of about 12% of the genes upregulated in M437[L] cells. This observation suggests that TFs may be involved in the regulation of L-A virus-related gene expression alterations and may be important for virus maintenance.

### 3.5. Role of Transcription Factors on Intracellular L-A dsRNA Content

Strains based on *S. cerevisiae* M437 and *S. paradoxus* AML-15-66 are prototrophic diploid yeasts; thus, genetic manipulations are challenging and labor-intensive. To determine the impact of TFs on the actual intracellular levels of the L-A virus genome, we employed laboratory *S. cerevisiae* yeast strains of BY4741 background. The parental *S. cerevisiae* BY4741 strain is haploid, natively harbors L-A-1 dsRNA, and is devoid of M satellite; thus, it is a non-killer strain. The selection of this genetic background enabled us to verify the relevance of observed transcriptional regulation patterns linked to the L-A virus in wild yeasts.

**Figure 6:**
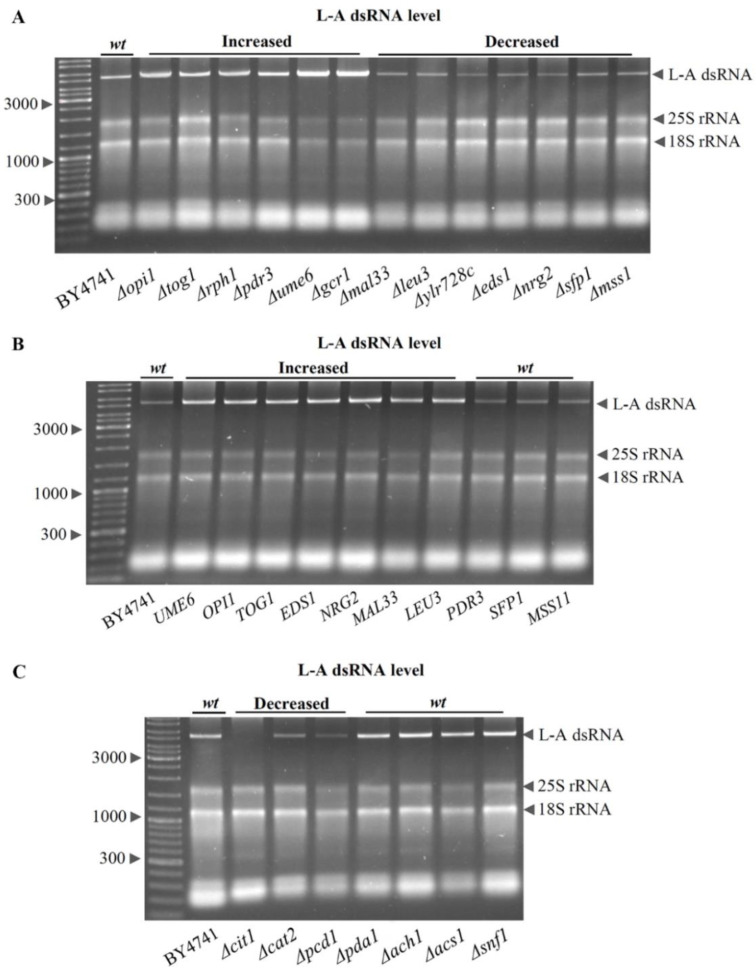
Relative L-A dsRNA content in *S. cerevisiae* cells upon the deletion or overexpression of single genes. Strains having increased or decreased amounts of L-A dsRNA are depicted; *wt* indicates levels similar to the parental BY4741 strain. The dsRNA levels of the L-A virus were normalized by 18S rRNA and compared to the *wt* control. L-A dsRNA levels in transcription factor (TF)-deletion strains (**A**), in strains overexpressing TF (**B**), and in strains lacking genes linked to the acetyl-CoA levels (**C**).

We selected the TFs listed in Figure 5 and tested how the elimination of each TF would affect the intracellular L-A dsRNA content in the BY4741 background. The single deletion of 13 TFs significantly altered L-A dsRNA levels (Figure 6A). The removal of two transcription activators (Δ*gcr1* and Δ*pdr3*) resulted in increased L-A levels, while the elimination of the other three (Δ*mss11*, Δ*sfp1*, and Δ*ylr278c*) led to decreased amounts of L-A dsRNA. The deletion of one out of four transcription inhibitors (Δ*opi1,* Δ*rph1,* Δ*tog1*, or Δ*ume6*) was linked to an increased L-A dsRNA content, while the elimination of the other four (Δ*eds1,* Δ*leu3,* Δ*mal33*, or Δ*nrg2*) led to a decreased L-A dsRNA content (Figure 6A). The removal of one of the other tested TFs did not lead to an observable change in the L-A dsRNA copy number (not shown); however, their role should not be neglected. Other TFs may have a more significant impact under other genetic backgrounds or growth conditions.

The overexpression effect of TFs, involved in significant L-A dsRNA content changes observed upon TF deletion, was analyzed. The overexpression of seven TFs (*EDS1, LEU3, MAL33*, *NRG2, OPI1, TOG1*, and *UME6*) led to higher amounts of L-A dsRNA (Figure 6B), whereas, in cells overexpressing *MSS11, PDR3*, or *SFP1* genes, the L-A dsRNA levels were similar to the wild type (Figure 6B). There was no decrease in L-A dsRNA in cells overexpressing any of the tested TFs, which may be related to galactose metabolism, since it was observed that more of L-A dsRNAs accumulate in cells grown on a nonfermentable carbon source [28,29].

Since L-A virus propagation requires the acetylation of the Gag protein [17,21,22], the impact of several genes linked to the regulation of the intracellular acetyl-CoA levels was analyzed (Figure 6C). In concordance with acetyl-CoA homeostasis being important for L-A virus propagation, the L-A dsRNA level was found to be decreased in Δ*cit1,* Δ*cat2,* and Δ*pcd1,* but not in Δ*pda1,* Δ*ach1,* Δ*acs1,* or Δ*snf1* strains.

## 4. Discussion

### 4.1. Gene Expression in Yeast Cells Maintaining Totiviridae L-A Virus

The adaptive modulation of host gene expression by the L-A virus likely evolved as a useful strategy for virus persistence and might result from long-term co-evolution. Consistent with previous findings [46,47,49], our data indicate that L-A dsRNA has a rather minor role in the host gene expression. Of the 450 DEGs in M437[L] cells, 328 were also differently expressed alike upon the elimination of solely L-A or both L-A and M dsRNAs, when compared to the wild-type killer cells [47], including the upregulation of *MAK10*, the essential gene for dsRNA replication [22]. Only two new DEGs (*MET2* and *SSA4*) were found in *S. paradoxus* AML-15-66 Pet[L] cells, since 35 genes were also differently expressed in cured Pet cells with respect to the wild-type killer cells [49]. Twenty-seven genes were also differently expressed in cured Pet cells, three genes (*FLO11*, *MET17,* and *HSP26*) were solely expressed in dsRNA-free Pet cells, and five genes (*MUP1, ACO1, IDH1, HIS4,* and *LYS20*) were only expressed in Pet cells possessing solely L-A dsRNA. Compared to previous observations, we conclude that it is not the helper L-A, but satellite M, virus that has a significant influence on the host transcriptome [47,49]. The satellite M virus not only employs L-A capsids for its genome replication, but it also utilizes the protein synthesis, maturation, modification, and secretion pathways of the yeast cell to promote killer toxin production and the maintenance of self-immunity [1,61,62,63]. Accordingly, killer toxin production is also associated with transcriptional responses linked to increased host cell stress tolerance [61].

Gene expression in Pet[L] cells seems to be the most distinct based on the number of genes affected and the relative alteration of gene expression. However, it is likely that this difference is also related to the respiration-deficient phenotype of Pet[L] cells [49]. *Petite* cells exhibit many physiological and metabolic differences in relation to the wild-type cells, including those with a small colony size, increased cell adhesion, low acetate catabolism, and inability to utilize nonfermentable carbon sources [64,65]. Notably, this phenotype might be advantageous, since it has been shown to promote heat resistance, to confer pleiotropic drug resistance, and even to prolong lifespan [64,66,67].

Interaction between the essential genes and L-A virus might be important for virus persistence. The essential role of mitochondria and iron–sulfur clusters in ribosome biogenesis [68] can be related to the strong upregulation of *FIT* genes, which is important for iron import in M437[L] cells. *MAK5* [69]*,* encoding for an essential nucleolar protein required for dsRNA maintenance, was also upregulated in M437[L] cells. This close relationship between the essential host genes and the virus may ensure that virus is transmitted to a healthy and viable host. It remains unclear why genes involved in mitochondrial gene expression and translation are downregulated in Spo[L] cells, although mitochondrial functions and proteins have been related with dsRNA maintenance. L-A virus replication is believed to occur in the cytoplasm; however, there is evidence that replicases of other RNA viruses colocalize with mitochondrial membranes; thus, mitochondria might also be related to the replication of the *Totiviridae* L-A virus [70]. The L-A virus-hosting Pet[L] strain is characterized by a reduction in genes prone to transcriptional alterations. This finding could provide further clues regarding the importance of yeast respiration in viral dsRNA maintenance. It would be interesting to determine if, at some point, the L-A virus becomes essential for yeast viability by driving the transcription of essential host genes. It remains to be determined if L-A viruses could help yeast to adapt to metabolic needs or other kinds of stress, or even provide an epigenetic memory for adaptive responses.

L-A-related transcriptional alterations are somewhat similar to those of other dsRNA mycoviruses. The infection of dimorphic fungi *Talaromyces marneffei* with dsRNA partitivirus TmPV1 resulted in the altered transcription of genes involved in the regulation of the transcription, transport, and metabolism of amino acids, lipids, and polysaccharides [71]. More viral genetic material was found in yeast than in the mycelial phase of *T. marneffei* and dsRNA enhanced the virulence of the fungi [71]. TmPV1 caused the suppression of RNA-interference genes in *T. marneffei*, suggesting that similar transcriptional reprogramming could have been performed by ancestral L-A dsRNA and contribute to the loss of RNA interference in many *Saccharomyces* and other yeast species [71,72]. Transcriptomic responses in totivirus-infected *Malassezia* yeasts were mostly related to ribosome biogenesis and other cellular processes [73,74]. The transcription of ribosomal RNA genes was also altered in *Sclerotinia sclerotiorum* fungus upon infection with *Hypoviridae* SsHV2-L dsRNA virus [75]. The importance of ribosomes and amino acids for the propagation of L-A dsRNA and its proteins has been documented [17,25]. It would be interesting to determine whether infection with L-A could contribute to yeast pathogenicity in symbiotic relationships with other living organisms, similarly to *Totiviridae Leishmania* virus 1 of the human pathogen *Leishmania braziliensis*, which is associated with the troublesome curing of leishmaniasis [76,77]. Our findings suggest that different host metabolic conditions can lead to distinct transcriptional responses in virus-infected and dsRNA-free cells. Targeting similar metabolic functions in other viral dsRNA-infected organisms may provoke a switch from harmless infection to pathogenesis [78]. It has already been demonstrated that virus-infected and virus-free yeasts show differences in their transcriptional responses to antiviral drugs [48].

The gene expression patterns induced by the L-A virus appear to be similar to those induced by various nutrient- and stress-related responses, and they are strongly linked to the host cell’s characteristics. The actual upregulation of genes encoding Bas1, Mig2, and Rsc30 TFs is consistent with the gene expression pattern of M437[L] cells. Mig2 and Rsc30 may be involved in upregulation and act as either direct or indirect activators of the expression of upregulated genes, whereas Bas1 may act as a transcriptional inhibitor and contribute to the observed downregulation of DEGs in cells infected with L-A virus. Bas1 is considered to control the response to a variety of stresses [79]. The Mig2 protein is involved in glucose-induced gene repression [58], and studies suggest that Mig2 plays a prominent role in the control of the yeast metabolic cycle and is linked to the mitotic cell cycle, metabolism of carbohydrates and amino acids, TCA cycle, endonuclease activity, endocytosis [80], and filamentous growth [81]. Rsc30 regulates the expression of ribosomal genes and the cell wall stress response [82]. The fact that we did not observe a significant overlap in TFs involved in the expression of the majority of DEGs in the different yeast species analyzed points to a complex and unique regulatory, transcriptional network for each yeast species [83], which is correspondingly adapted to the co-evolution of the virus and its host cell.

### 4.2. Interconnection between Transcription Factors and L-A dsRNA Levels

**Figure 7:**
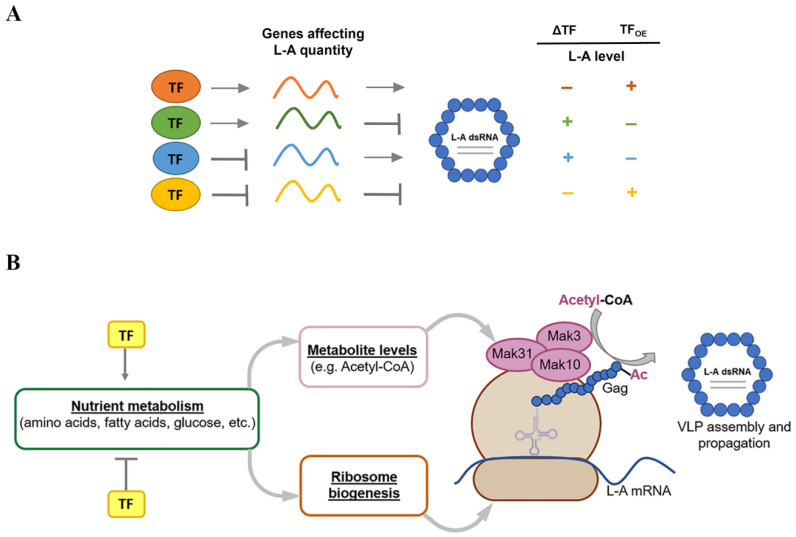
Schematic representation of links between transcription factors (TFs), differently expressed genes (DEGs), and levels of L-A virus. (**A**) TF can either activate (orange and green TFs) or inhibit (blue and yellow TFs) the transcription of genes. DEGs can either favor or repress the propagation of L-A virus, thereby resulting in alterations of the L-A dsRNA levels. Consequently, the deletion (ΔTF) or overexpression (TF_OE_) of certain TFs can contribute to changes in gene expression. The elimination or overexpression of certain TFs can change—increase (+) or decrease (−)—the intracellular L-A dsRNA level by changing the balance of transcripts that are important for L-A virus maintenance. All TFs act simultaneously to impact the total amount of L-A virus. (**B**) Relationship between TFs and known cellular factors affecting L-A virus replication. TFs regulate the transcription of genes involved in nutrient metabolism, which is directly linked to the cellular metabolite levels and ribosome biogenesis. Acetyl-CoA is a substrate for the NatC acetyltransferase complex, composed of Mak3, Mak10, and Mak31 subunits, which is necessary for co-translational Gag protein acetylation. The translation of viral proteins by ribosome and Gag acetylation is essential for L-A virus assembly and propagation.

We hypothesize that some DEGs in L-A-maintaining cells are either virus-favoring or virus-inhibiting, and transcriptional regulation is maintained mainly by cellular TFs (Figure 7A). We assumed that higher expression of virus-favoring genes may increase the amount of intracellular L-A dsRNAs, while the expression of virus-inhibiting genes could result in decreased virus propagation. We propose that L-A virus’ persistence is accompanied by increased expression of genes favoring and genes inhibiting L-A maintenance (Figure 7A). Numerous TFs are involved in this complex regulatory network. The virus might act towards the activation of genes required for its replication (e.g., *MAK3, MAK10,* and *MAK31* [17,21,22]), whereas the host may induce the expression of genes involved in virus suppression (e.g., *XRN1/SKI1, SKI2, SKI3, SKI7, SKI8, NUC1,* and *POR1/2* [28,33,44,45]) to control virus propagation and vice versa. Similarly, the virus may contribute to the repression of the transcription of genes conflicting with its functioning; similarly, the host might inhibit the expression of genes that aid the virus. The balance of regulation must be met to optimize the functioning of the virus–host system. Although the regulation of gene expression in yeasts is complex and is not limited to TFs (reviewed in [84,85]), in our model, we simplified the regulation of gene transcription. We assumed that TFs directly or indirectly regulate the transcription of genes, linked to the dsRNA amount in the L-A virus (Figure 7A). We predicted the possible effect on the L-A dsRNA levels if TF involved in any type of transcriptional regulation is eliminated (ΔTF) or overexpressed (TF_OE_).

Our data support the hypothesis based on the effects of both elimination and overexpression for the role of at least four TFs, namely Eds1, Leu3, Mal33, and Nrg2. All of these TFs act in a way to significantly increase the amount of L-A virus, likely by lowering the expression of virus-inhibiting genes (corresponding to the yellow TF in Figure 7A). In Reimand et al.’s study [86], Eds1 and Mal33 were associated with the transcriptional repression of *POR1*, which is known to negatively affect the L-A copy number [28], confirming that our results are consistent with previous findings. Other TFs affecting virus propagation are also associated with the transcriptional regulation of genes linked to the L-A virus levels; however, we did not observe a lower L-A dsRNA amount in cells upregulating any of the tested TFs (Figure 6B). This indicates the eminent importance of carbon metabolism in the regulation of the L-A levels, particularly in the absence of glucose.

Out of the 13 TFs that significantly contributed to the L-A dsRNA levels, nine TFs were linked with glucose metabolism, two TFs were associated with ribosome biogenesis (Rph1, Sfp1, and YLR278C), and one was associated with amino acid biosynthesis (Leu3).

Sfp1 activates the expression of ribosomal protein genes and is responsive to nutrients and stress [87]. Sfp1 could act in all groups of TFs in our model (Figure 7A), based on the published data. Sfp1 was associated with the positive regulation of the expression of the *MAK10* gene, which is beneficial for L-A virus maintenance [88] (Figure 7A, orange TF), but also of genes linked to L-A virus repression (Figure 7A, green TF), namely *NUC1*, *POR1*, and *SKI2* [86,89]. Along this line, Sfp1 is associated with the inhibition of *MAK3* expression [86], which is required for L-A propagation (Figure 7A, blue TF), and of *POR2,* and *SKI7* [86,90]—genes linked to L-A virus repression (Figure 7A, yellow TF). Interestingly, Sfp1 regulates its own gene transcription, in addition to other genes coding for TFs involved in significant alterations of the L-A levels, namely Eds1, Nrg2, Pdr3, Rph1, and Gcr1 [86,90,91].

Seven TFs were associated with similar alterations in the L-A dsRNA levels, namely decreasing upon TF deletion, and increasing or exhibiting no change upon TF overexpression (Figure 7), such as *Sef1*. The elimination or overexpression of *NRG2, EDS1*, and *LEU3*, results in decreased or increased levels of L-A dsRNA. Nrg2 mediates glucose repression and a group of stress-response genes [92]. The function of Eds1 is suggested by its paralog Rgt1, a glucose-responsive TF regulating glucose transport [93]; its transcription is not only regulated by Sfp1, but also by Leu3 [86]. Leu3 regulates the transcription of genes involved in the biosynthesis of branched-chain amino acids [94]. Mal33 and Mss11 are both related to the regulation of carbohydrate metabolism [95,96], and their elimination results in a decreased amount of L-A virus (Figure 7A). Mss11 is also involved in cellular signaling in response to nutrient levels [96]. The function of *YLR278C* is unknown, but it is localized in the nucleus and a certain part of the *YLR278C*-encoded protein enhances the formation of [URE3] prion, and is also involved in the regulation of the yeast metabolic cycle and chromatin remodeling [80,97].

Six TFs were linked to increased L-A dsRNA levels upon TF elimination or TF overexpression, except for *PDR3*, the overexpression of which resulted in the wild-type L-A level (Figure 7). Most of these TFs are related to glucose; Tog1 activates gluconeogenesis genes and non-fermentable carbon metabolism [98], Gcr1 activates glycolysis genes [99], and Ume6 is a glucose-dependent repressor [100]. Opi1 is required for normal cardiolipin levels and mitochondrial function [101], whereas Pdr3 responds to mitochondrial protein import stress and is involved in multidrug resistance signaling [102]. Rph1 suppresses the genes of ribosome biogenesis in response to nutrient levels [103].

These observations suggest that TFs, changing the level of the L-A virus, may act via the regulation of ribosome biogenesis, glucose-dependent gene expression, and the acetyl-CoA level (Figure 7B). Rph1 and Sfp1 appear to be the main TFs regulating ribosome biogenesis and the quantity of L-A virus. Consistent with the requirement of ribosomes for the translation and co-translational acetylation of viral proteins, Δ*rph1* cells lack the repression of ribosome biogenesis and have more L-A dsRNA. However, Δ*sfp1* lacks activation of ribosome biogenesis genes and leads to a decreased virus level (Figure 6A). Glucose represses *MAK10* expression [30]; thus, it may negatively regulate the Gag protein acetylation and assembly of L-A particles. We suggest that acetyl-CoA is the central metabolite that determines not only the efficacy of L-A virus propagation, but also the energetic state of the cell. It is involved in the regulation of gene expression through the acetylation of histones, TFs, and enzymes, and affects cellular metabolism and growth (reviewed in [104]). The generation of acetyl-CoA occurs in the mitochondrial and nucleo-cytosolic compartments of the cell, where the catabolism of glucose, fatty acids, and branched-chain amino acids is a major driver [104].

Based on this idea, we aimed to analyze the L-A virus abundance in cells that have genetic defects related to acetyl-CoA turnover (Figure 6C). Interestingly, we observed a decrease in the amount of L-A viral genome in a Δ*cit1* mutant strain lacking mitochondrial citrate synthase. This mutant strain is believed to contain a higher level of mitochondrial acetyl-CoA due to its inability to use acetyl-CoA for citrate synthesis [105]. As Gag protein acetylation is believed to occur in cytoplasm, a decrease in the quantity of L-A dsRNA in Δ*cit1* strain may be linked to the increased acetylation of mitochondrial proteins [105], or to impaired citrate synthesis, as citrate is needed as a metabolite for cytoplasmic acetyl-CoA production. Along this line, lower L-A abundance was detected in Δ*cat2* strain, consistent with the disruption of acetyl-CoA transport into the cytoplasm in a form of acetyl-carnitine from peroxisomes in this mutant [105]. The elimination of *PDC1* resulted in diminished L-A levels, likely due to the lower capacity of β-oxidation in peroxisomes and lower generation of acetyl-CoA therein [106]. The L-A levels in other mutants tested did not change, suggesting that a subunit of the pyruvate dehydrogenase Pda1, acetyl-CoA hydrolase/transferase Ach1, and acetyl-CoA synthetase Acs1 had no significant impact on L-A propagation in glucose medium (Figure 6C). We also tested the role of protein kinase Snf1, which is required for proper glucose-dependent gene expression and cellular acetyl-CoA levels [107,108]. Even though Δ*snf1* was reported to have about 70% less acetyl-CoA than the wild-type strain [108], the L-A level in this mutant was not altered. Current methods do not allow for the quantification of acetyl-CoA levels in distinct cellular compartments; thus, it remains a challenging task to determine the relationship between cytosolic acetyl-CoA level and L-A maintenance.

The crystal structure of the yeast NatC complex has been recently resolved, and a high efficiency for L-A Gag N-terminus acetylation was observed, pointing to the evolutionary adaptation of the virus [109]. There is a relatively low number of yeast proteins that are acetylated by the NatC complex in comparison to the other acetyltransferases; however, only three mitochondrial proteins (namely Kgd1, Fum1, and Mrp1) are known to have identical N-termini to the Gag protein and are acetylated in a similar manner [110]. The NatC complex is evolutionarily conserved from yeast to humans, but the genetic background is important for complementation, and differences are believed to be linked to mitochondrial function and stress resistance [111]. In line with acetyl-CoA being a central intermediate of the pathways required to metabolize non-fermentable carbon sources [104], the disruption of the NatC complex results in reduced yeast growth on non-fermentable carbon sources [23,28,111]. N-terminal acetylation is thought to be an irreversible modification, and the alteration of this process is linked to the development of disease in humans [112,113]. The function and substrates of NatC complex subunits need to be clarified.

The regulatory associations between TFs and L-A virus propagation require more investigation. The cell and TFs do not discriminate between genes affecting the L-A levels per se. The same TFs likely control both types of genes and have overlapping functions or even opposite regulation patterns depending on the environmental factors and active cellular metabolism pathways. This insight helps to comprehend why, upon L-A virus infection in one strain, certain genes are upregulated, while they are downregulated in the other. We also observed this contradiction with the TFs Yrr1, Mga2, and Sut1, since they were associated with both the up- and downregulation of DEGs in different strains (Figure 5). Yrr1 is associated with the pleiotropic drug resistance pathway [114]. The Mga2 protein is involved in the response to hypoxia, cobalt ion treatment, and iron ion deprivation [115,116]. Sut1 regulates various processes (sterol uptake [117], hypoxic gene expression [118], and the repression of filamentation-inducing genes [119]) in response to pheromones [120]. The elimination of the majority of tested TFs did not significantly change the L-A dsRNA level, probably due to the long-lasting coevolution of the host and virus establishing the self-compensating transcriptional landscape.

## 5. Conclusions

The persistence of L-A dsRNA leads to an adaptive transcriptional response in *S. cerevisiae* and *S. paradoxus* hosts. The least impact of the L-A virus on the host gene expression was observed in the respiration-deficient (*S. paradoxus* Pet[L]) strain, while the most changes were induced in *S. cerevisiae* (M437[L]) cells. The majority of DEGs were related to the biosynthesis of amino acids and ribosomes, including a part of the essential host genes. Altered transcription patterns were similar to those induced upon various nutrient and stress responses and dsRNA-virus infections in fungi. We have revealed the role of several yeast transcription factors predicted to regulate gene expression in L-A-maintaining cells based on experimental data and our model. The elimination of most of the tested transcription factors did not result in a significant change in the L-A genome levels; however, Δ*gcr1,* Δ*opi1,* Δ*pdr3,* Δ*rph1,* Δ*tog1*, or Δ*ume6* resulted in an increased L-A dsRNA amount, while Δ*eds1,* Δ*leu3,* Δ*mal33*, or Δ*nrg2* resulted in a decreased L-A dsRNA amount in *S. cerevisiae* cells. The overexpression of genes encoding the transcription factors Eds1, Leu3, Mal33, Nrg2, Opi1, Tog1, and Ume6 led to increased L-A genome levels in *S. cerevisiae* cells grown in galactose medium. The elimination of *CIT1, CAT2,* and *PCD1* genes resulted in reduced intracellular L-A viral genome levels, further indicating an important role of host cell acetyl-CoA-related metabolism viral maintenance. The yeast respiration mode is yet another factor regulating the transcriptional response to the presence of L-A virus in *Saccharomyces paradoxus*. We observed significant species- and metabolism-oriented specificity in alterations of the transcriptional landscape stemming from the presence of the L-A virus. Taken together, our findings further advance our understanding of the adaptive mechanism employed by widespread *Totiviridae* viruses to promote viral propagation in yeast cells.

## Data Availability

Not applicable.

## Supplementary Materials

The following supporting information can be downloaded at: https://www.mdpi.com/article/10.3390/jof8040381/s1, Table S1: Differently expressed genes and GO terms of altered transcription genes in M437[L], Spo[L], and Pet[L] cells. Essential genes are indicated by color; Table S2: Transcription factors affecting the expression of differently expressed genes in M437[L], Spo[L], and Pet[L] cells.
